# Neurological and inflammatory biomarkers in CSF along the Alzheimer's Disease spectrum

**DOI:** 10.1002/alz70856_097201

**Published:** 2025-12-24

**Authors:** Catherine Demos, Jermaine Brown, Brian Ngo, Itziar de Rojas, Federico Casales, Victoria Fernández, Josep Blazquez, Miyo K Chatanaka, Pilar Sanz, Sergi Valero, Mercè Boada, Eleftherios P Diamandis, Martin Stengelin, Anu Mathew, George Sigal, Xavier Morató Arús, Jacob N Wohlstadter

**Affiliations:** ^1^ Meso Scale Diagnostics, LLC., Rockville, MD, USA; ^2^ Ace Alzheimer Center Barcelona‐Universitat Internacional de Catalunya, Barcelona, Spain; ^3^ Networking Research Center on Neurodegenerative Diseases (CIBERNED), Instituto de Salud Carlos III, Madrid, Spain; ^4^ Ace Alzheimer Center Barcelona‐Universitat Internacional de Catalunya, Barcelona, Barcelona, Spain; ^5^ University of Toronto, Toronto, ON, Canada; ^6^ Sinai Health System, Toronto, ON, Canada

## Abstract

**Background:**

Alzheimer's disease (AD) is a heterogeneous neurodegenerative disease with a decades‐long prodromal period. Monitoring of sequential pathological changes in neurodegeneration, inflammation, neurovascular dysfunction, oxidative stress and metabolic stress may provide the opportunity for intervention before symptom onset. Assessment with multiple biomarkers may also inform more tailored therapeutic intervention.

**Methods:**

Using MULTI‐ARRAY technology, 54 biomarkers were measured using less than 200 μL of CSF from individuals with AD dementia (*n* = 100), mild cognitive impairment (MCI) with progression to dementia during the following 3‐year follow‐up (*n* = 100), MCI non‐progressors (*n* = 100), and subjective cognitive decline (SCD) (*n* = 93), collected by ACE Alzheimer Center Barcelona. Biomarkers were selected to cover multiple putative disease mechanisms such as neurovascular dysfunction, inflammation, neurodegeneration, tissue injury, and metabolic stress. One‐way ANOVA with Bonferroni correction was applied to determine groupwise differences. Area under the curve (AUC) for receiver operating characteristic curves was calculated to assess biomarker utility for predicting dementia progression.

**Results:**

For 43 assays, more than 80% of samples provided concentrations within the dynamic range of the assay. We found concentration differences of 30 CSF biomarkers to be statistically significant across cognitive groups, with the most significant groupwise comparisons between the dementia groups (AD and MCI progressors) and the non‐dementia groups (MCI non‐progressors and SCD). There were 17 analytes for which mean comparisons were statistically different between MCI progressor and non‐progressor groups. Ten proteins, pTau217, total tau, neurofilament light, GFAP, MIF, MMP‐10, YKL‐40, neurofilament heavy, MIP‐1α, and IL‐15, demonstrated an AUC > 0.7 for differentiation of MCI progressors and non‐progressors, showing promise for differentiating MCI individuals at risk of progressing to dementia, with ptau217 being the most significant (AUC > 0.99).

**Conclusions:**

Here we present an exploratory study with quantitative immunoassays, where we identified several CSF biomarkers indicative of dementia or progression to dementia covering multiple pathological mechanisms. Further successful integration into a biomarker panel could help personalize treatment, stratify individuals for therapeutic studies and provide a better understanding of how these early pathologies impact disease progression.

